# Analysing the origin of long-range interactions in proteins using lattice models

**DOI:** 10.1186/1472-6807-9-4

**Published:** 2009-01-29

**Authors:** Orly Noivirt-Brik, Ron Unger, Amnon Horovitz

**Affiliations:** 1 Department of Structural Biology, Weizmann Institute of Science, Rehovot 76100, Israel; 2 The Mina & Everard Goodman Faculty of Life Sciences, Bar-Ilan University, Ramat-Gan, 52900, Israel

## Abstract

**Background:**

Long-range communication is very common in proteins but the physical basis of this phenomenon remains unclear. In order to gain insight into this problem, we decided to explore whether long-range interactions exist in lattice models of proteins. Lattice models of proteins have proven to capture some of the basic properties of real proteins and, thus, can be used for elucidating general principles of protein stability and folding.

**Results:**

Using a computational version of double-mutant cycle analysis, we show that long-range interactions emerge in lattice models even though they are not an input feature of them. The coupling energy of both short- and long-range pairwise interactions is found to become more positive (destabilizing) in a linear fashion with increasing 'contact-frequency', an entropic term that corresponds to the fraction of states in the conformational ensemble of the sequence in which the pair of residues is in contact. A mathematical derivation of the linear dependence of the coupling energy on 'contact-frequency' is provided.

**Conclusion:**

Our work shows how 'contact-frequency' should be taken into account in attempts to stabilize proteins by introducing (or stabilizing) contacts in the native state and/or through 'negative design' of non-native contacts.

## Background

There is a wealth of information that indicates that distant sites in proteins are often coupled to each other energetically. Evidence for such coupling initially emerged through studies of allosteric regulation of proteins [1] when it became clear that allosteric control is often achieved by ligand binding-induced conformational changes that are propagated from one ligand binding site to other distant sites. Later, it became possible to identify distant sites in proteins that are coupled to each other energetically by protein engineering through the use of the double-mutant cycle (DMC) method [for review see ref. [2]]. It has become clear from many such DMC studies that distant sites in proteins are often coupled to each other in a weak but significant manner [for review see ref. [3]]. More recently, it has become possible to demonstrate long-range coupling experimentally also by employing NMR methods [4]. Finally, computational methods have also indicated the presence of long-range communication in proteins. One class of computational methods is based on detection of co-evolving residues in multiple sequence alignment data. Such methods were originally developed in order to detect residues that are in physical contact [5,6] but, more recently, have been used to reveal long-range pathways of energetic connectivity in proteins [7-9]. Long-range communication in proteins has also been revealed in computational studies based on normal mode analysis and its coarse-grained versions in which correlations between fluctuations of distant residues are detected [10-13].

Despite the wealth of evidence indicating that long-range communication is extremely common in proteins, the physical basis of this phenomenon is still unclear. In addition, there are some uncertainties associated with many of the computational and experimental methods used to detect such long-range interactions. For example, it is not clear whether correlated mutations at distant positions reflect long-range coupling or common ancestry [14-17]. In the case of the DMC method, there is always a concern that the calculated coupling energy reflects a reorganization energy in one or more of the mutants in the cycle and not the true pairwise interaction energy [18]. Given these reasons, we decided to explore whether long-range interactions exist in 2D and 3D lattice models of proteins although such interactions are not an input feature of them. Simple lattice models of proteins have proven to capture some of the basic properties of real proteins and, although they ignore many important details, they have been used successfully for elucidating general principles of protein folding and stability [19-26]. Here, we show by invoking computational DMC analysis that long-range interactions are also common in lattice models of proteins. Hence, our results indicate that long-range communication in proteins may also occur as a result of interactions in the non-native states and not just *via *pathways by which information is transmitted through the native state structure as other computational methods suggest [7,12]. Our analysis also shows that the values of the coupling energies of both short- and long-range interactions have a linear dependence on their respective contact frequencies in the conformational ensemble.

### Theory

The energy of a sequence in a specific lattice conformation, E(C), is calculated by summing all the pairwise contact energies, e_ij _(see Table 1), between neighboring lattice points excluding consecutive residues in the sequence, as follows:

**Table 1:** Pairwise residue interaction energies.

	H	P	+	-	B
H	-1	0	0	0	0

P	0	-0.75	-0.25	-0.25	0

+	0	-0.25	+1	-1.25	0

-	0	-0.25	-1.25	+1	0

B	0	0	0	0	0

The interaction energies (e_ij_) between pairs of residues in contact reflect in a qualitative manner the strengths of interactions between different types of amino acids: hydrophobic (H), neutral polar (P), positively charged (+), negatively charged (-) and blank (B) for the use of mutations.

(1) $$\text{E}(\text{C})=\sum_{\text{j}>\text{i}+2}^{\text{N}}{\text{e}}_{\text{ij}}\delta (\left|{\text{r}}_{\text{i}}-{\text{r}}_{\text{j}}\right|)$$

where |r_i _- r_j_| is the distance in lattice units between residues i and j that are separated in sequence by at least two residues and $\delta (x)=\left\{ \begin{array}{l} \begin{array}{cc} 1 & x=1 \end{array}\\ \begin{array}{cc} 0 & otherwise \end{array} \end{array}\right.$. The free energy of folding, Δ*G*, of the native conformation of a sequence was calculated using [21]:

(2) $$\Delta G=-kT\ln(\frac{P_{N}}{1-P_{N}})$$

where *P*_N _is the probability that the chain is in its native state. This probability is given by: $P_{N}=\frac{e^{-E(N)/kT}}{Q}$, where $\text{Q}=\sum_{\text{C}\in \text{Z}}{\text{e}}^{-\text{E}(\text{C})/kT}$ (Z is the ensemble of all possible conformations on the relevant lattice), *E*(N) is the energy of the native conformation, *T *is the temperature and *k *is the Boltzmann constant. Eq. (2) can be written as follows: $\Delta G=-kT\ln\left(\frac{{\text{e}}^{-\text{E}(\text{N})/kT}}{Q-{\text{e}}^{-\text{E}(\text{N})/kT}}\right)$. It, therefore, follows that:

(3) Δ*G *= *E*(N) + *kT*ln(*Q *- e^-E(N)/*kT*^)

We designate the sum over all the non-native conformations by *Q*' where *Q*' = *Q *- e^-E(N)/*kT*^.

The strength of a pairwise interaction can be estimated from DMC calculations or by computing the perturbation energy, ΔΔ*G*_per _= Δ*G*_wt _- Δ*G*_m_, where Δ*G*_wt _and Δ*G*_m _are the respective free energies of the wild-type native conformation before and after a particular pairwise interaction is removed ('turned off') without affecting any other interactions. For simplicity, the derivation that follows is for this measure termed 'perturbation energy' and not for the coupling energy calculated from DMC that involves more algebraic terms (see Methods). It is important to note, however, that the perturbation energy of a pairwise interaction is almost equal to the coupling energy calculated from DMC for that interaction since in the DMC method the effects of the different mutations on other interactions tend to cancel out [18]. We show in the Results that our derivation holds for perturbation energies as well as for coupling energies that, in contrast with the perturbation energies, can be determined in experiments. The perturbation energy can be expressed, as follows:

(4) ΔΔ*G*_per _= *E*_c _- *kT*ln(*Q*'_m_/*Q*'_wt_)

where E_c _is the energy of the contact that was removed. It is convenient to partition the sum of all the non-native conformations of the mutant, Q'_m_, into the sets of C_1 _and C_2 _conformations (|C_1_| + |C_2_| = N) in which the interaction being targeted is either absent or present, respectively, as follows: Q'_m _= $\sum_{\text{C}\in {\text{C}}_{\text{1}}}{\text{e}}^{-\text{E(C)/}kT}+\sum_{\text{C}\in {\text{C}}_{2}}{\text{e}}^{-\text{(E(C)}-\lambda )/kT}$, where *λ *is the contact energy of the perturbed interaction (Table 1). The expression for Q'_m _can be rewritten, as follows:

$\begin{array}{c} {\text{Q'}}_{\text{m}}=\sum_{\text{C}\in {\text{C}}_{\text{1}}}{\text{e}}^{-\text{E(C)/}kT}+\sum_{\text{C}\in {\text{C}}_{2}}{\text{e}}^{-\text{E(C)/}kT}({\text{e}}^{\lambda /kT}+1-1)\\ =\sum_{\text{i}=1}^{\text{N}}{\text{e}}^{-{\text{E}}_{\text{i}}/kT}+\sum_{\text{C}\in {\text{C}}_{2}}{\text{e}}^{-\text{E}(\text{C})/kT}({\text{e}}^{\lambda /kT}-1) \end{array}$ .

Eq.(4) can, therefore, be rewritten as:

(5) $$\begin{array}{c} \Delta \Delta G_{\text{per}}=E_{\text{c}}-kT\ln(\frac{\sum_{\text{i}=1}^{\text{N}}{\text{e}}^{-{\text{E}}_{\text{i}}/kT}+({\text{e}}^{\lambda /kT}-1)\sum_{\text{C}\in {\text{C}}_{2}}{\text{e}}^{-\text{E}(\text{C})/kT}}{\sum_{\text{i}=1}^{\text{N}}{\text{e}}^{-{\text{E}}_{\text{i}}/kT}})\\ =E_{\text{c}}-kT\ln(1+\frac{({\text{e}}^{\lambda /kT}-1)\sum_{\text{C}\in {\text{C}}_{2}}{\text{e}}^{-\text{E}(\text{C})/kT}}{\sum_{\text{i}=1}^{\text{N}}{\text{e}}^{-{\text{E}}_{\text{i}}/kT}}) \end{array}$$

Taylor series expansion (ln(1+x) ≈ x for |*x*| < 1) of Eq. (5) and multiplication of the resulting expression by $\frac{1}{\text{Q}|\text{Z}|}/\frac{1}{\text{Q}|\text{Z}|}$ (= 1) yields:

(6) $$\begin{array}{c} \Delta \Delta G_{\text{per}}=E_{\text{c}}-\frac{kT({\text{e}}^{\lambda /kT}-1)\sum_{\text{C}\in {\text{C}}_{2}}{\text{e}}^{-\text{E}(\text{C})/kT}}{\sum_{\text{i}=1}^{\text{N}}{\text{e}}^{-{\text{E}}_{\text{i}}/kT}}\\ =E_{\text{c}}-\frac{kT(({\text{e}}^{\lambda /kT}-1)/|\text{Z}|)\sum_{\text{C}\in {\text{C}}_{2}}\frac{{\text{e}}^{-\text{E}(\text{C})/kT}}{\text{Q}}}{\text{Q’/Q|Z|}} \end{array}$$

The Boltzmann weighted contact frequency, BWCF(i, j), is defined as: $\left(\sum_{\text{C}\in \text{Z}}\frac{e^{-\text{E}(\text{C})/\text{kT}}}{\text{Q}}\delta ({\left|{\text{r}}_{\text{i}}-{\text{r}}_{\text{j}}\right|}_{\text{c}}))/|\text{Z}\right|$, where i and j are two positions in the sequence and each occurrence of a contact is multiplied by the Boltzmann weight of the conformation (C) in which it occurs. Hence, inspection of Eq. (6) shows that plots of the perturbation energy (or coupling energy) as a function of BWCF(i, j) are expected to be approximately linear with a slope that is a function of λ.

## Results and discussion

**Figure 1:**
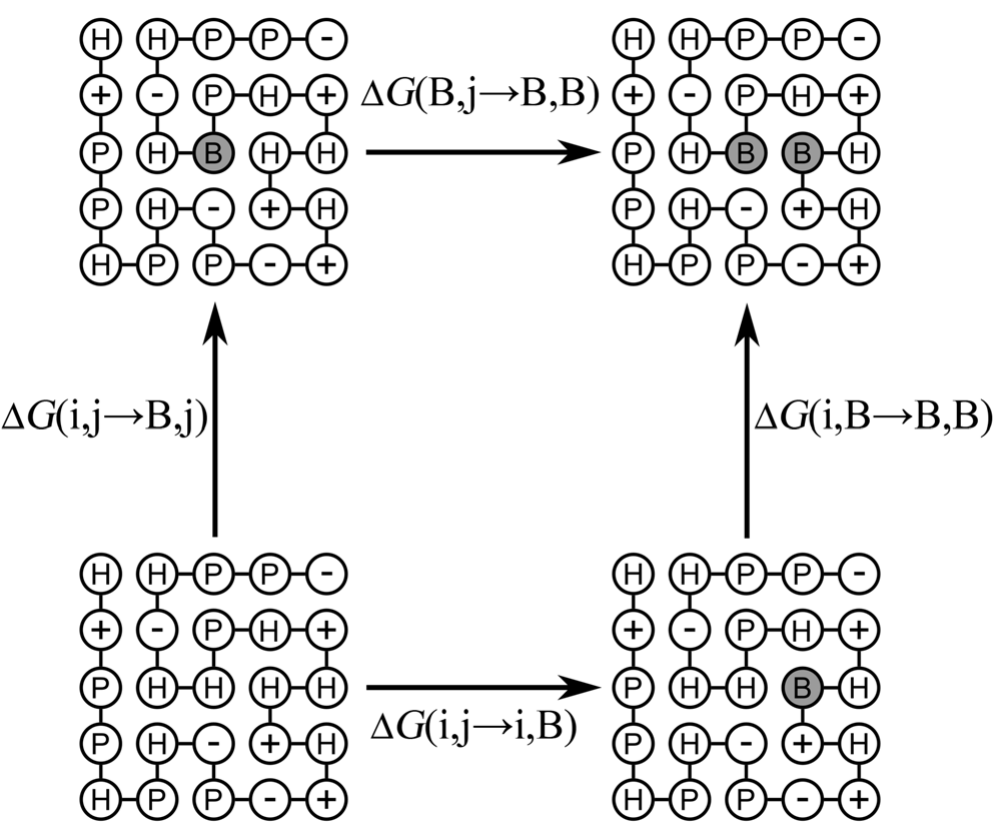
**Scheme of a double-mutant cycle for a 2D lattice model protein**. Two residues, i and j, are mutated (the mutations are designated by B on a dark background) either singly or in combination. Δ*G*(i,j→B,j) and Δ*G*(i,B→B,B) are the respective free energy changes upon mutation of residue i when residue j is present and when it has also been mutated. If these free energy changes are equal to each other then residues i and j are not coupled. Otherwise, residues i and j are energetically coupled. The same is true for the difference between the free energy changes Δ*G*(i,j→i,B) and Δ*G*(B,j→B,B). In this scheme, residues i and j form a direct contact in the native structure of the wild-type sequence. The double-mutant cycle method can be applied, however, also for residues that are distant in space in the native structure as carried out in the paper.

**Figure 2:**
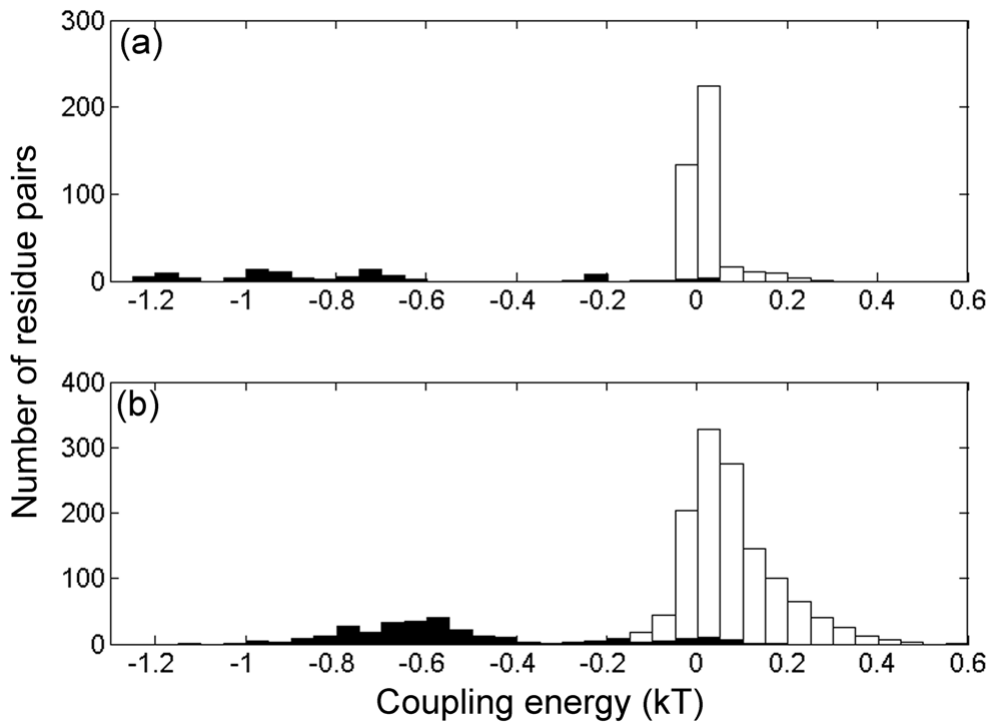
**Distributions of the values of the pairwise coupling energies for all possible pairs of positions in sequences with different native states on 2D and 3D lattices**. The values of the coupling energies for all possible pairs of positions in 10 sequences of 16 residues with different native states on a 2D lattice with full enumeration (a) and 10 sequences of 27 residues with different native states on a 3 × 3 × 3 3D lattice (b) were calculated. The distributions of the values of the pairwise coupling energies for positions in contact and not in contact in these native conformations are shown by filled and empty bars, respectively.

DMC have been used extensively to determine experimentally the strengths of various pairwise interactions in proteins [2]. Here, DMC were invoked in order to evaluate, for the first time to the best of our knowledge, coupling energies between all possible pairs of positions in 2D and 3D lattice models of proteins (Figure 1). Evidence for correlations between distant sites in lattice models has been reported before in the context of protein aggregation [27]. The distributions of the values of the coupling energies for all possible pairs of positions in the different native states of 10 sequences with 16 residues on a 2D lattice with full enumeration and 10 sequences with 27 residues on a 3 × 3 × 3 3D lattice are shown in Figure 2a and 2b, respectively. It can be seen that the values of the coupling energies for pairs in contact are mostly negative whereas the values of the coupling energies for pairs that are not in contact are mostly (but not exclusively) positive and smaller in absolute terms. Pairs that are in contact in a given native conformation could, therefore, be identified with high confidence using this procedure.

**Figure 3:**
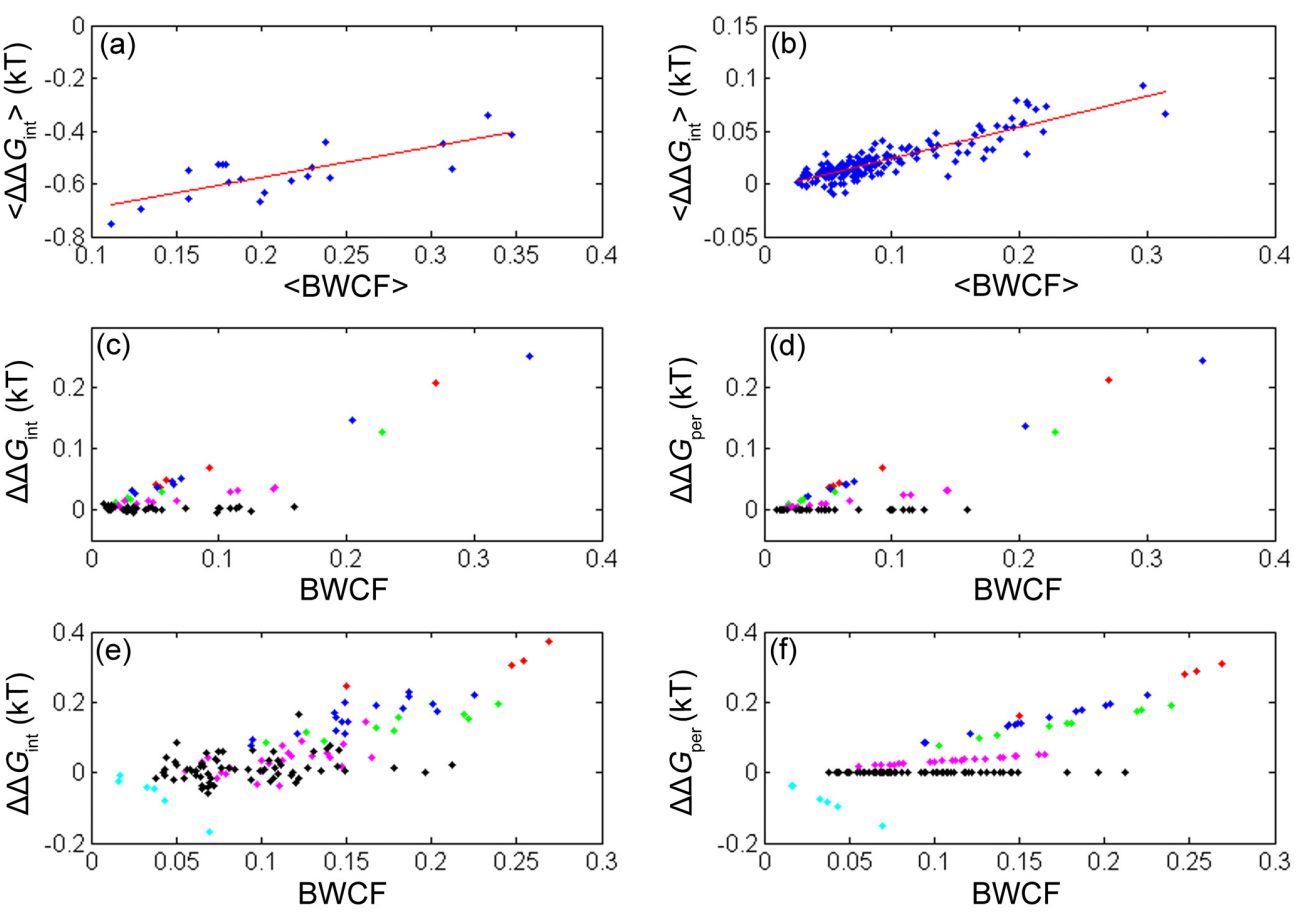
**Plots of different measures of the strength of pairwise interactions as a function of measures of contact frequency for several representative examples of lattice models**. In panels a and b, the average coupling energies, <ΔΔ*G*_int_>, of all the pairs in contact (a) and not in contact (b) are plotted against their respective average BWCF in the case of a set of sequences with 30 residues that have the same native conformation on a lattice of 5 × 6. In panels c and d, the coupling (c) and perturbation (d) energies, ΔΔ*G*_int _and ΔΔ*G*_per_, are plotted against the BWCF for all the pairs of positions not in contact in the case of a sequence with L = 20 on a 2D lattice with full enumeration. In panels e and f, the coupling (e) and perturbation (f) energies are plotted against the BWCF for all the pairs of positions not in contact in the case of a sequence with L = 27 on a 3 × 3 × 3 cubic lattice. The data in panels c-f corresponding to different values of λ are color coded, as follows: λ = -1.25, red; λ = -1, blue; λ = -0.75, green; λ = -0.25, magenta; λ = 0, black; λ = 1, cyan.

The fraction of conformations in the ensemble in which residues at two positions in a sequence are in contact is termed the 'contact frequency'. The 'contact frequency' is not defined for pairs of consecutive positions in a sequence since the interaction energy of such pairs is by definition zero (see Eq. (1)). It is also not defined for pairs of even or odd positions in a sequence since they cannot interact on a square or cubic lattice and, thus, have a contact-frequency of zero. Therefore, only pairs of residues with non-zero values of contact-frequency are considered here. A more accurate measure of the frequency of a contact in a conformational ensemble is the 'Boltzmann weighted contact frequency', BWCF, where the occurrence of each contact is multiplied by the Boltzmann weight of the conformation (C) in which it is found (see Theory). In the Theory section it was shown that the strength of a pairwise interaction is expected to have a linear dependence on its BWCF. Such linear plots of different measures of the strength of pairwise interactions as a function of BWCF are depicted in Figure 3 for several representative examples of lattice models.

In the first example (Figure 3a and 3b), a set of sequences with a length, L, of 30 residues that have the same native structure was constructed (such structure-based sequence sets are designated SBSS) and the coupling energy was determined for every possible pair of positions in each sequence. The average value of the coupling energy for each pair of positions in the SBSS was then calculated in order to improve the signal-to-noise ratio. In this example, only conformations that fit into a 5 × 6 lattice were considered. It may be seen that a strong linear correlation is found between the average coupling energy for each pair of positions in the SBSS and the corresponding average BWCF index. This correlation holds for pairs of residues that form native contacts (Figure 3a, *r *= 0.78; *P*-value = 5.5 × 10^-5^) and also, surprisingly, for pairs of residues that are not in contact in this particular native conformation (Figure 3b, *r *= 0.87; *P*-value = 1.3 × 10^-55^). Such linear correlations (with average correlation coefficients of about 0.84 (± 0.05) for the non-contacting pairs and 0.62 (± 0.15) for the pairs in contact) were also found for SBSS that correspond to 8 other native conformations (i.e. 2 SBSS for sequences with L = 30 on a 5 × 6 lattice, 4 SBSS for sequences with L = 25 on a 5 × 5 lattice and 2 SBSS for sequences with L = 25 on a 5 × 6 lattice) when only the conformations that fit into the lattice were considered.

In the second example, the coupling (Figure 3c) and perturbation (Figure 3d) energies for all residue pairs not in contact in the native state of a sequence with L = 20 on a 2D lattice are plotted as a function of their BWCF. Here, values of the BWCF were calculated for the entire conformational ensemble (|Z| = 41,889,578) and not just for the relatively compact states as in Figure 3a and 3b. The color-coding designates the different contacts that have a given value of λ (Table 1). It may be seen (Figure 3d) that almost perfect correlations (*r *≈ 1) are found between the perturbation energies and the BWCF for each given value of λ as predicted by Eq. (6). The correlations between the coupling energies and the BWCF for each given value of λ (except for λ = 0) are also excellent (Figure 3c, *r *> 0.97; P-value < 10^-6^) but not perfect as those in Figure 3d for the perturbation energies. Plots for residue pairs in contact in the native state are not shown since the number of such pairs is small and the correlations are, thus, not significant.

In the third example, the coupling (Figure 3e) and perturbation (Figure 3f) energies for all residue pairs not in contact in the native state of a sequence with L = 27 on a 3 × 3 × 3 lattice are plotted as a function of their BWCF. Here, too, almost perfect correlations (*r *≈ 1) are found between the perturbation energies and the BWCF for each given value of λ (Figure 3f) whereas the correlations for the coupling energies (Figure 3e) are excellent (*r *= 0.92, 0.85, 0.92, 0.58 and 0.97 for λ values of -1.25, -1, -0.75, -0.25 and 1, respectively, with *P*-values < 2 × 10^-3 ^except for λ = -1.25 where the number of data points, n, is small (n = 4)) but not perfect as those in Figure 3f. In summary, therefore, the data depicted in Figure 3 for different types of lattice models (2D or 3D lattices with or without full enumeration of all the conformational states in the ensemble and for single sequences or averaged for a SBSS) support the general result described by Eq. (6) that the free energies of both direct (in contact in the native state) and indirect pairwise interactions are linearly dependent on their Boltzmann-weighted contact frequencies. It should be pointed out that only weak or no correlations are observed when pairwise energies taken directly from Table 1 are plotted against the BWCF, thereby providing further justification for the approach in this study that is based on the coupling or perturbation energies. The correlations in Figure 3 indicate that rare native contacts have more negative coupling energies than abundant native contacts. Likewise, rare non-contacting pairs have less positive coupling energies than abundant non-contacting pairs. Therefore, one may infer that native states can be stabilized by stabilizing contacts with low contact-frequency and destabilizing non-contacting pairs with a high contact-frequency.

Given that the interaction energy of a sequence in a specific lattice conformation is calculated by summing over all pairwise interactions between neighboring lattice points, it may seem surprising that non-direct interactions with significant positive coupling energies are found to exist (Figure 3). However, it has been pointed out that the strengths of pairwise interactions in the native state determined by DMC are always relative to the unfolded state [28]. Hence, the positive coupling energies observed here in the case of non-contacting pairs reflect, to a large extent, pairwise interactions in the non-native conformations in the ensemble. Surprisingly, however, positive coupling energies are also observed in the case of residue pairs such as P, H that have interaction energies of zero (Table 1) and, therefore, should not be coupled even when they are in contact in non-native conformations. These non-zero coupling energies arise owing to non-additivity in entropy calculations [29].

The correlations shown in Figure 3 can be understood more intuitively by considering several extreme cases and keeping in mind that the free energy of the native state is a function of both the energy of the native conformation and the energies of all the other non-native conformations in the ensemble (see Eq. (3)). For simplicity, the Boltzmann weights of the different states will be neglected in the discussion that follows and we will, therefore, refer to the contact-frequency (and not the BWCF) of residue pairs. The following four extreme cases of perturbations will be considered: (i) elimination of a native contact with a contact-frequency of 1/|Z|; (ii) elimination of a native contact with a contact-frequency that approaches one; (iii) elimination of a non-native contact with a contact frequency of 1/|Z|; and (iv) elimination of a non-native contact with a contact-frequency that approaches one.

**Figure 4:**
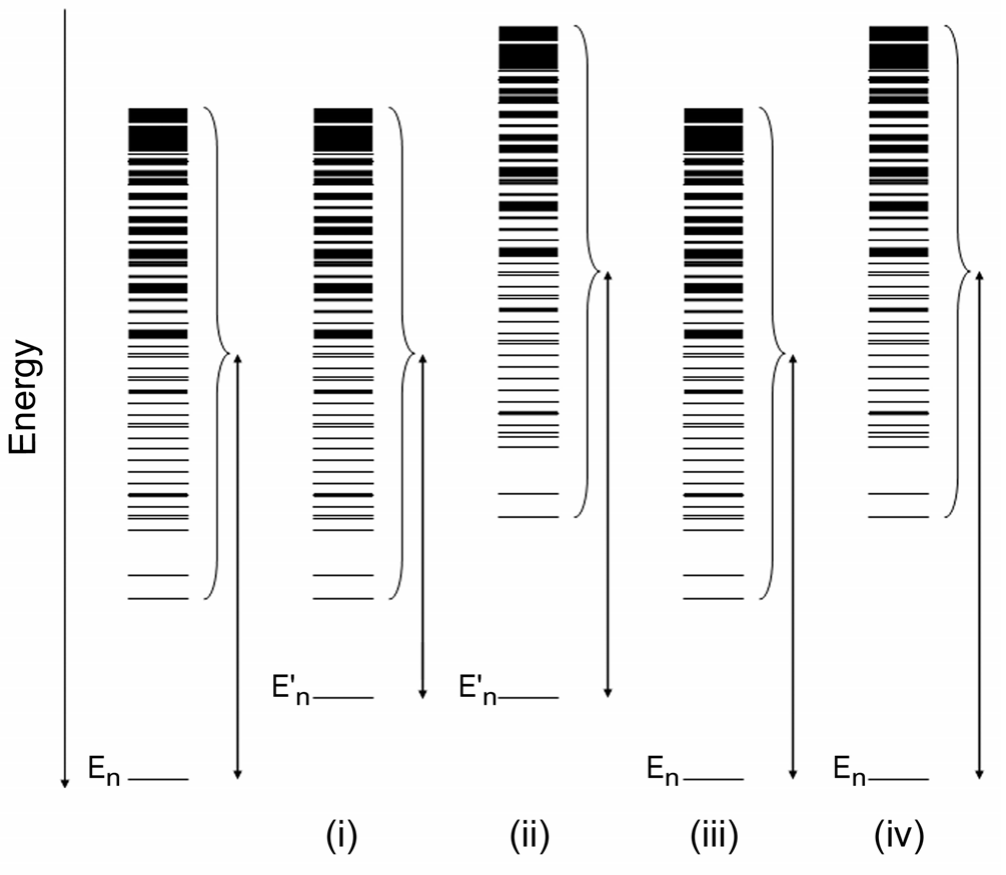
**Effects of different perturbations on the energy spectrum of the native state and the ensemble of non-native conformations**. The effects of four different extreme cases of perturbations are depicted. In case (i), a native contact with a contact-frequency of 1/|Z| (|Z| is the ensemble size) is eliminated, thereby causing the energy of the native state, E_n_, to increase to E'_n _but not affecting the energies of the non-native states. The gap between the energy of the native state and the energies of all the non-native states is, therefore, reduced by E'_n_-E_n_. In case (ii), a native contact with a contact-frequency value that approaches one is eliminated, thereby causing the energies of the native state and most of the non-native states to increase by E'_n_-E_n _without changing the energy gap. In case (iii), a non-native contact with a contact frequency of 1/|Z| is eliminated without changing the energy gap as there is no change in the energies of the native state and most of the non-native states. In case (iv), a non-native contact with a contact-frequency value that approaches one is eliminated, thereby increasing the energies of most of the non-native states and also the gap in energy between these states and the native state.

In the first case, a contact that exists only in the native state is perturbed and, therefore, only the energy of the native state is affected. Hence, the gap between the energy of the native conformation and the energies of the non-native conformations is reduced (Figure 4, case (i)). Such a perturbation reduces Δ*H *by the value of the contact energy, *E*_c_, and has no effect on Δ*S *(which is a function of the sum, Q', over all the non-native states). The perturbation energy, ΔΔ*G*_per_, in this case is, therefore, equal to *E*_c_.

In the second case, a contact that exists in both the native state and in most of the non-native states is perturbed and, therefore, the gap between the energy of the native conformation and the energies of the non-native conformations hardly changes (Figure 4, case (ii)). In this case, *Q*'_m_/*Q*'_wt _< 1 and the perturbation energy, ΔΔ*G*_per _= *E*_c _- *kT*ln(*Q*'_m_/*Q*'_wt_), therefore, increases (note that *E*_c _is negative) in accordance with the plot in Figure 3a. Native contacts with a low contact-frequency, therefore, contribute more than those with a large contact-frequency to the gap between the energy of the native state and the energies of the non-native conformations, thereby explaining why they have more negative coupling energies (Figure 3a).

In the third case of a perturbation of a non-native contact with a low contact frequency, it is clear that the energies of the native state and most of the non-native states do not change and, therefore, the energy gap also remains unchanged (Figure 4, case (iii)). In the fourth case of a perturbation of a non-native contact with a high contact-frequency, most of the non-native conformations are destabilized but the energy of the native state is not affected and the gap between the energy of the native conformation and the energies of the non-native conformations, therefore, becomes larger (Figure 4, case (iv)). In cases such as (iii) and (iv), when a pairwise interaction between residues that are not in contact in the native state is removed, there is no effect on Δ*H *and the perturbation energy is given by: ΔΔ*G*_per _= - *kT*ln(*Q*'_m_/*Q*'_wt_). If the contact-frequency of the removed interaction is low (case (iii)), then *Q*'_m _≈ *Q*'_wt _and the perturbation energy will be equal to approximately zero. If the contact-frequency of the removed interaction is high (case (iv)), then *Q*'_m_/*Q*'_wt _< 1 and the value of the perturbation energy will increase in accordance with the plots in Figure 3. Non-native contacts with a high contact-frequency, therefore, contribute more than those with a low contact-frequency to the gap between the energy of the native state and the energies of the non-native conformations, thereby explaining why they have more positive coupling energies (Figure 3). The effects shown schematically in Figure 4 almost always result in an increase of the energy of either the native state (case (i)), the non-native states (case (iv)) or both (case (ii)) since non-favorable pairwise interactions (Table 1) are rare given the amino acid composition we used. It is clear, however, that protein evolution might favor non-favorable interactions in non-native conformations that would destabilize them relative to the native state. Such an evolutionary process termed 'negative design' [30-32] would be reflected in negative (favorable) coupling energies between residues that are not in contact in the native state.

**Figure 5:**
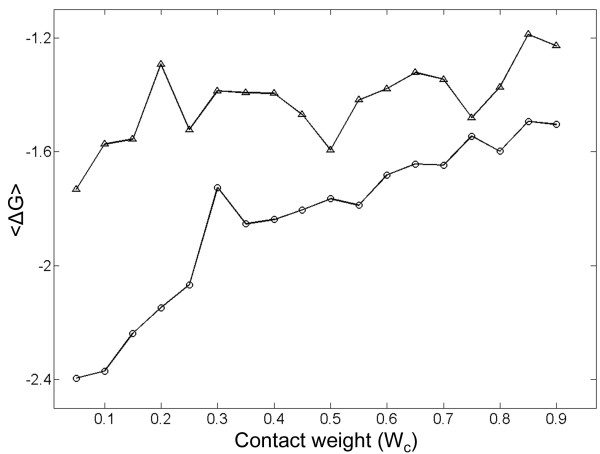
**Stabilization of 2D-lattice model proteins by taking into consideration the contact frequency of residue pairs in contact and not in contact in the native state**. The average free energy of folding of 100 sequences designed either with (○) or without (△) taking into account the contact frequency is plotted against the value of the contact weight, W_c _(see Eqs. (8) and (9)), used in the design. The results shown here are for sequences with L = 30 on a 5 × 6 lattice. Similar results were obtained in cases of other lattice dimensions and sequence lengths when only the most compact conformations were considered. For more details, see Methods.

How important is contact-frequency for protein stability? In order to obtain some insight into this question, we compared the stabilization achieved when optimizing a sequence for a particular native conformation using two different functions: (i) F1 (Eq. (8)) that minimizes the energy of native contacts and maximizes the energy of non-native contacts ('negative design'); and (ii) F2 (Eq. (9)) in which the contributions of native and non-native contacts is weighted by their contact-frequency. Both functions have an adjustable parameter, W_c_, which determines the relative weight of the contributions of the native vs. non-native interactions to stability. It can be seen (Figure 5) that for sequences with L = 30 on a 5 × 6 lattice, greater stability is achieved when contact-frequency is taken into account across the entire range of W_c _values. Similar results were obtained in cases of other lattice dimensions and sequence lengths when only the most compact conformations were considered. A more general scoring function will be needed for efficient design when the entire conformational space is considered.

## Conclusion

It is shown in this study that long-range pairwise interactions are also present in simple lattice models of proteins despite the fact that the interaction energy of a sequence in a specific conformation is based solely on direct interactions (Eq. (1)). Double-mutant cycle analysis of these lattice models and a mathematical analysis show that the strength of both direct and indirect native interactions increases (i.e. their coupling free energy becomes more negative) in a linear fashion with decreasing contact-frequency that is an entropic term. Hence, proteins can be stabilized by introducing (or stabilizing) contacts in the native state with a low contact-frequency and removing (or destabilizing) contacts in non-native states with a high contact-frequency, as shown in Figure 5. Although manifestations of the latter strategy of 'negative design' have been recognized before [32] it has not been fully appreciated how the choice of interactions to be introduced (stabilized) or removed (destabilized) affects the extent of stabilization. Our findings are not dependent on sequence length and lattice dimensions that determine the conformational ensemble size and are, thus, likely to be relevant to the selection of folding pathways, folding rates and the design of real proteins. It may be possible to implement our findings using ensembles that are derived computationally (such as with COREX [33]) before experimentally characterized conformational ensembles become available. The new approach described here, that involves combining DMC analysis with lattice models, may also pave the way for a rigorous analysis of other complex aspects of protein behavior. For example, simulation of protein evolution by subjecting lattice models to rounds of mutagenesis followed by selection can be used to assess the contribution of correlated mutations at distant positions to protein folding, stability and allosteric communication. Employing lattice models to address this issue has the distinct advantage that it renders possible separating between correlated mutations due to common ancestry and those due to biophysical factors. Such studies may reveal relationships between contact-frequency, correlated mutations and other properties of proteins such as contact-order [34].

## Methods

### The lattice model of proteins

2D or 3D lattice models that are similar to the one described by Jacob and Unger [35] were used. In brief, the protein sequence consists of an alphabet of five amino acids: hydrophobic (H), neutral polar (P), positively charged (+), negatively charged (-) and blank (B) for the use of mutations. The pairwise interaction energies (e_ij_) are taken from Table 1 and reflect in a qualitative manner the strengths of interactions between different types of amino acids. Similar results were obtained using other contact interaction matrices. The energies of all possible conformations of a given sequence on a particular lattice were calculated and the conformation with the lowest energy, if a single such one exists, was considered as its native conformation. A value of 1 was used for *kT*. It is important to note that the size of the ensemble, |Z|, is determined by the lattice dimensions and the same conformation of a given sequence may, therefore, have different values of Δ*G *due to different lattice dimensions.

Sequences of length (L) 16, 20, 25 and 30 were used for the 2D models and sequences with L = 27 for the 3D models. In the case of sequences with L = 16 or 20, all the respective 802,075 and 41,889,578 non-symmetric conformations were enumerated. In the case of sequences with L = 25 or L = 30 where the total number of conformations is too large to enumerate, we considered only the conformations that could be fitted into 5 × 5 or 5 × 6 lattices. Likewise, only the conformations that could be fitted into a 3 × 3 × 3 lattice were considered in the case of the 3D lattice models for sequences with L = 27. The numbers of all compact non-symmetric conformations of sequences with L = 25 on 5 × 5 and 5 × 6 lattices are 1081 and 377,779, respectively. The numbers of all compact non-symmetric conformations of sequences with L = 30 on a 5 × 6 lattice and L = 27 on a 3 × 3 × 3 lattice are 6431 and 103,346, respectively. The sequences were generated by random rearrangements of L residues with compositions of 44% H, 31% P, 12.5% (+) and 12.5% (-) in the case of sequences with L = 16, 40% H, 28% P, 16% (+) and 16% (-) in the case of sequences with L = 25, 42% H, 30% P, 14% (+) and 14% (-) in the case of sequences with L = 30 and 40% H, 30% P, 15% (+) and 15% (-) in the case of sequences with L = 20 or 27 (these compositions correspond roughly to those in the PDB).

### Generation of structure-based sequence sets (SBSS)

SBSS that contained more than 40 different sequences of the same length and with the same native conformation were generated. These SBSS have a mean sequence identity that is only between 0.29–0.34 since (as described above) the sequences were generated by random rearrangements and, thus, represent a random sample of sequence space. Nine different SBSS corresponding to different native conformations were examined.

### Calculation of coupling energies using double-mutant cycles

The strength of a pairwise interaction between residues i and j in the native conformation of a given sequence was evaluated by constructing a DMC that comprises the original wild-type sequence, two single mutants in which either residue i or j are replaced with the blank (B) residue and the corresponding double mutant in which both residues are replaced with this residue. The blank residue corresponds to alanine which is usually chosen as a reference state in experimental DMC since it is assumed that (i) replacement by this residue tends, in general, to reduce structural perturbations upon mutation and that (ii) interactions between alanine at one position and any other type of residue at the second position are minimal. The coupling energy, ΔΔ*G*_int_, which is a measure of the strength of the pairwise interaction between residues i and j was calculated, as follows:

(7) ΔΔ*G*_int _= Δ*G*_i,j _- Δ*G*_i,B _- Δ*G*_B,j _+ Δ*G*_B,B_

where Δ*G*_i,j_, Δ*G*_i,B_, Δ*G*_B,j _and Δ*G*_B,B _are the respective free energies of folding of the wild-type protein, the two single mutants and the double mutant that are calculated using Eq. (2). The coupling energy is equal to the difference in the free energies of two parallel processes in the cycle, Δ *G*(i,j→B,j) and Δ*G*(i,B→B,B), that correspond to the effect of mutating residue i (or j) when the other residue is present or absent, respectively (Figure 1). In these calculations, negative and positive coupling energies reflect interactions that stabilize or destabilize the native state, respectively. We implemented such an experiment for each given pair of positions so that a coupling energy could be calculated for every possible pair of positions in each sequence.

### Calculation of perturbation energies

We also calculated a perturbation energy, ΔΔ*G*_per_, = Δ*G*_wt _- Δ*G*_m_, for every possible pair of positions in each sequence where Δ*G*_wt _and Δ*G*_m _are the respective free energies of the wild-type native conformation before and after a particular pairwise interaction is 'turned off' but without affecting any other interactions. Under ideal circumstances [18], the coupling energy, which can be determined experimentally or calculated as described above, provides a good estimate of the perturbation energy that can only be determined by computation.

### Contact frequency-based protein stabilization

Sequences with a specific native conformation were generated by a Monte Carlo (MC) process that maximizes two design scores, F_1 _and F_2_, that either ignore the contact frequency or take it into account, respectively. The expressions for the scores are:

(8) $${\text{F}}_{1}={\text{W}}_{\text{c}}\frac{1}{{\text{N}}_{\text{c}}}\sum_{\text{c}}{\text{e}}^{-{\text{E}}_{\text{C}}}+(1-{\text{W}}_{\text{c}})\frac{1}{{\text{N}}_{\text{non}}}\sum_{\text{non}}{\text{e}}^{+{\text{E}}_{\text{non}}}$$

(9) $${\text{F}}_{2}={\text{W}}_{\text{c}}\frac{1}{{\text{N}}_{\text{c}}}\sum_{\text{c}}(1-{\text{f}}_{\text{c}}){\text{e}}^{-{\text{E}}_{\text{C}}}+(1-{\text{W}}_{\text{c}})\frac{1}{{\text{N}}_{\text{non}}}\sum_{\text{non}}{\text{f}}_{\text{c}}{\text{e}}^{+{\text{E}}_{\text{non}}}$$

where W_c _is the contact weight, N_c _and N_non _are the total number of contacts and non-contacts in the specific conformation, respectively, and f_c _is the contact-frequency. The values of W_c_ were varied between 0.05–0.95. For each value of W_c_, 100 designed sequences were generated in 10,000 MC steps and the average free energy of folding was then calculated.

## Authors' contributions

ON carried out all the calculations. AH wrote the paper. All the authors analysed the data, helped draft the paper and read and approved the final manuscript.
